# Enhanced heterogeneous Fenton-like degradation of methylene blue by reduced CuFe_2_O_4_†

**DOI:** 10.1039/c7ra12488k

**Published:** 2018-01-03

**Authors:** Qingdong Qin, Yahong Liu, Xuchun Li, Tian Sun, Yan Xu

**Affiliations:** School of Civil Engineering, Southeast University Nanjing 210096 China xuxucalmm@seu.edu.cn +86 25 83790757 +86 25 83790757; School of Environmental Science and Engineering, Zhejiang Gongshang University Hangzhou 310018 China

## Abstract

To facilitate rapid dye removal in oxidation processes, copper ferrite (CuFe_2_O_4_) was isothermally reduced in a H_2_ flow and used as a magnetically separable catalyst for activation of hydrogen peroxide (H_2_O_2_). The physicochemical properties of the reduced CuFe_2_O_4_ were characterized with several techniques, including transmission electron microscopy, X-ray diffraction, X-ray photoelectron spectroscopy and magnetometry. In the catalytic experiments, reduced CuFe_2_O_4_ showed superior catalytic activity compared to raw CuFe_2_O_4_ for the removal of methylene blue (MB) due to its relatively high surface area and loading Fe^0^/Cu^0^ bimetallic particles. A limited amount of metal ions leached from the reduced CuFe_2_O_4_ and these leached ions could act as homogeneous Fenton catalysts in MB degradation. The effects of experimental parameters such as pH, catalyst dosage and H_2_O_2_ concentration were investigated. Free radical inhibition experiments and electron spin resonance (ESR) spectroscopy revealed that the main reactive species was hydroxyl radical (˙OH). Moreover, reduced CuFe_2_O_4_ could be easily separated by using an external magnet after the reaction and remained good activity after being recycled five times, demonstrating its promising long-term application in the treatment of dye wastewater.

## Abstract 2

CuFe_2_O_4_ was reduced for activation of hydrogen peroxide and the reduced CuFe_2_O_4_ showed a relatively higher catalytic activity.

## Graphical abstract

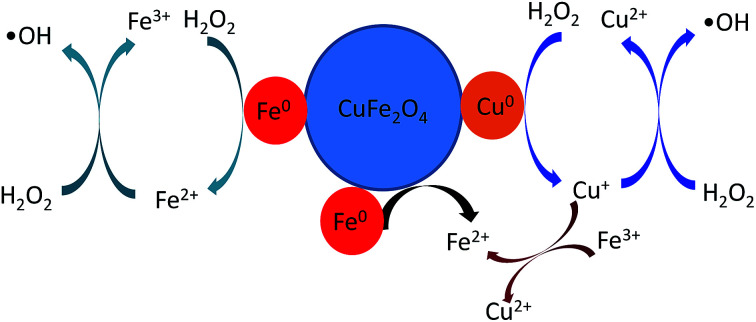

## 1. Introduction

The textile industry produces a large amount of wastewater that is extremely harmful to humans and the environment since it contains a high concentration of dyes and a variety of recalcitrant organic compounds. Therefore, many treatment processes such as physical separation, chemical oxidation and biological degradation have been widely employed for the removal of dyes from wastewater.^1^ Among the different water treatment approaches tested so far, advanced oxidation processes (AOPs) have shown great potential for the treatment of industrial wastewaters.^2^ AOPs are characterized by hydroxyl radical (˙OH) with a redox potential of 2.80 eV, which can react with almost all recalcitrant organic compounds. As one of the most effective AOPs, Fenton process has unique advantages due to its simple generation of ˙OH by a reaction between Fe^2+^ and H_2_O_2_, low cost and environmental benignity.^3^ However, the application of traditional homogeneous Fenton processes is limited by the requirement of low solution pH (<4) and the formation of ferric hydroxide sludge during wastewater treatment. Therefore, alternative catalysts for the Fenton reaction are pursued to overcome the aforementioned drawbacks of the Fe^2+^/H_2_O_2_ system.

Recently, heterogeneous Fenton-like processes have been investigated as a more practical and efficient alternative technique for removing recalcitrant organic pollutants.^4^ Many iron based catalysts, such as Fe_2_O_3_,^5^ Fe_3_O_4_,^6^ α-FeOOH^7^ and CuFe_2_O_4_ ^8,9^ have been applied to activate H_2_O_2_ into reactive radicals for the degradation of organic pollutants in water. In particular, CuFe_2_O_4_, a kind of magnetic material with cubic structure, has received considerably higher attention in water treatment due to its high magnetic permeability, excellent chemical and mechanical stability.^10^ Feng *et al.* prepared CuFe_2_O_4_ nanoparticles as a heterogeneous Fenton-like catalyst to degrade sulfanilamide and found that the pseudo-first-order rate constant was 5.9 × 10^−3^ min^−1^.^8^ Wang *et al.* synthesized mesoporous CuFe_2_O_4_ as a heterogeneous Fenton-like catalyst to degrade imidacloprid and reported that the apparent reaction rate constant was 1.7 × 10^−2^ min^−1^.^9^ However, CuFe_2_O_4_ seems to present weak catalytic activity due to its low electron transfer rate, which limits the practical application of heterogeneous Fenton-like catalyst.

Zero-valent Fe (Fe^0^) has proved to be an efficient catalyst for the heterogeneous Fenton-like reaction due to the generation of ferrous iron by the corrosion of metal iron.^11,12^ In addition, Fe^0^ as an electrons donor could reduce Fe^3+^ to Fe^2+^, which could be able to accelerate the formation of ˙OH.^13^ Nevertheless, Fe^0^ trends to aggregate and forms large particles due to strong anisotropic dipolar interactions, which leads to a decrease in surface area and ultimately a lower catalytic activity.^14^ Therefore, it is essential to anchor and immobilize Fe^0^ onto supports to prevent aggregation. Several studies have supported Fe^0^ on Fe_3_O_4_ surface to enhance organic compounds degradation.^13–16^ These results showed a significant increase in activity for the oxidation of organics due to a thermodynamically favorable electron transfer from Fe^0^ to Fe_3_O_4_ producing active Fe^2+^ species. More recently, to achieve better catalytic activity, iron–copper bimetallic catalyst system has also attracted increasing attention.^17,18^ The combination of copper with iron exhibits an improved catalytic activity due to the synergic effects of two-metal redox couples. For instance, Wang *et al.* synthesized iron–copper bimetallic nanoparticles embedded within ordered mesoporous carbon (CuFe–MC) and observed a greater catalytic activity of CuFe–MC than those of Fe–MC and Cu–MC.^17^ The reactions in Fenton-like system with iron–copper bimetallic nanoparticles were described by the following equations:^17,18^

1

<svg xmlns="http://www.w3.org/2000/svg" version="1.0" width="23.636364pt" height="16.000000pt" viewBox="0 0 23.636364 16.000000" preserveAspectRatio="xMidYMid meet"><metadata>
Created by potrace 1.16, written by Peter Selinger 2001-2019
</metadata><g transform="translate(1.000000,15.000000) scale(0.015909,-0.015909)" fill="currentColor" stroke="none"><path d="M80 600 l0 -40 600 0 600 0 0 40 0 40 -600 0 -600 0 0 -40z M80 440 l0 -40 600 0 600 0 0 40 0 40 -600 0 -600 0 0 -40z M80 280 l0 -40 600 0 600 0 0 40 0 40 -600 0 -600 0 0 -40z"/></g></svg>

Fe^0^ + 2H^+^ → Fe^2+^ + H_2_

2 Fe^0^ + H_2_O_2_ + 2H^+^ → Fe^2+^ + 2H_2_O

3 2Cu^0^ + H_2_O_2_ + 2H^+^ → 2Cu^+^ + 2H_2_O

4 Fe^2+^ + H_2_O_2_ → Fe^3+^ + ˙OH + OH^−^

5 Fe^3+^ + H_2_O_2_ → Fe^2+^ + ˙O_2_H + H^+^

6 Cu^+^ + H_2_O_2_ → Cu^2+^ + ˙OH + OH^−^

7 Cu^2+^ + H_2_O_2_ → Cu^+^ + ˙O_2_H + H^+^

8 Fe^3+^ + Cu^+^ → Fe^2+^ + Cu^2+^

9 Fe^0^ + 2Fe^3+^ → 3Fe^2+^

Therefore, to increase the catalytic activity of CuFe_2_O_4_, the surface modification of CuFe_2_O_4_ by introducing Fe^0^ and Cu^0^ was proposed in this study.

In this work, we used H_2_ to reduce CuFe_2_O_4_ and obtained zero-valent iron–copper bimetallic particles on the surface of CuFe_2_O_4_. The reduced CuFe_2_O_4_ was then used as Fenton-like catalyst. The overarching goal of this study was to develop a powerful candidate of heterogeneous Fenton-like catalyst. Methylene blue (MB) was selected as a model compound for dyes. The common influencing parameters on MB degradation were comprehensively investigated. The magnetic separation and regeneration of reduced CuFe_2_O_4_ were performed. Finally, the possible catalytic mechanism was also discussed.

## 2. Materials and methods

### 2.1. Materials

H_2_O_2_ (30%, w/w) was of analytical grade and was supplied by Sinopharm Chemical Reagent Co. (Shanghai, China). Other chemicals (analytical grade) used in the study were purchased from Sigma-Aldrich and used without further purification. All solutions were prepared using 18 MΩ deionized H_2_O at neutral pH (Millipore, USA). The stock solutions containing 250 mg L^−1^ of MB were freshly prepared by dissolving appropriate amounts of MB and kept in the dark.

### 2.2. Preparation of reduced CuFe_2_O_4_

The CuFe_2_O_4_ was synthesized in classical alkaline medium using conventional literature recipes.^19^ In brief, 0.025 mol CuCl_2_·2H_2_O and 0.05 mol FeCl_3_·6H_2_O were dissolved together in 100 mL of deionized H_2_O, and then 75 mL NaOH solution (4 M) was added dropwise, followed by heating at 90 °C. The black precipitate was homogenized by vigorous stirring for 2 h at 90 °C and then washed by deionized water several times, until the water pH did not change. Finally, the CuFe_2_O_4_ was filtrated and dried at 70 °C overnight followed by calcination in flowing air at 400 °C for 4 h.

The reduced CuFe_2_O_4_ was prepared by thermal treatment at 400 °C in a quartz tube under H_2_ (99.99%) flow (30 mL min^−1^) for 4 h with a heating rate of 10 °C min^−1^. After reduction, the material was cooled down under H_2_ flow to room temperature and was transferred to a sample vial and kept sealed under nitrogen atmosphere prior to use.

### 2.3. Characterization

Transmission electron micrograph (TEM) of the samples was taken on a Hitachi H-8100 TEM, operated at 200 kV. Powder X-ray diffraction (XRD) patterns were recorded on a Philips PW1710 diffractometer using Cu Kα radiation. Nitrogen adsorption–desorption isotherms were measured at 77 K on a Micromeritics ASAP 2020 sorptometer, with the samples outgassed for 16 h at 110 °C and 10^−6^ Torr prior to measurement. X-ray photoelectron spectroscopy (XPS) of the above mentioned samples were recorded on a spectrometer (Perkin-Elmer PHI-5300/ESCA, USA) with an Al Kα X-ray source. All the binding energies were referenced to the neutral C 1s peak at 284.6 eV to compensate for the surface charging effects. The XPS results were collected as binding energy forms and fitted using a curve-fitting program (XPSPEAK41 software).

### 2.4. Experimental procedure

Batch degradation experiments of MB were carried out in a 100 mL conical flask at 25 °C in the dark. The initial concentration of MB was 50 mg L^−1^, and the total volume of reaction solution was 50 mL. The reaction suspension was prepared by adding the required amount of catalyst into 50 mL solution that had been adjusted to the desired pH value by 0.1 M HNO_3_. A known concentration of H_2_O_2_ was added to the solution to initiate the reaction. Samples were taken at set intervals using a 1 mL syringe, and quenched with excess methanol. To evaluate the contribution of homogeneous Fenton catalyzed by the leaching Fe and Cu ions on the MB degradation, experiment was carried out as follows: after mixing reduced CuFe_2_O_4_ at solution pH 3.2 for 25 min and removing the catalyst by filtration, MB and H_2_O_2_ were then added into the filtrate. The reusability of the catalyst was evaluated by collecting the catalyst with a magnet, washing with deionized water, drying the used catalyst under vacuum, and using it for the next reaction under similar experimental conditions. The catalytic activities were calculated by the concentration of MB (*C*/*C*_0_), where *C*_0_ and *C* were the MB concentrations at initial and time *t*, respectively.

## 3. Results and discussion

### 3.1. Characterization

**Fig. 1:**
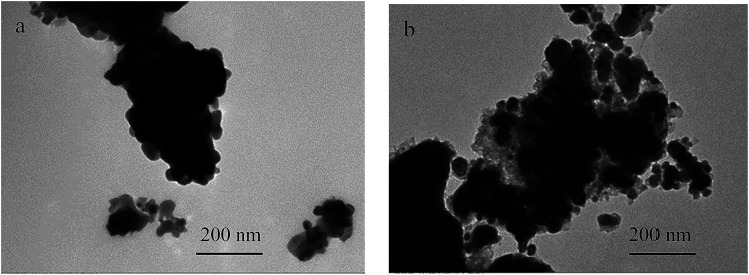
TEM images of (a) CuFe_2_O_4_ and (b) reduced CuFe_2_O_4_.

The transmission electron microscope (TEM) images of the CuFe_2_O_4_ and reduced CuFe_2_O_4_ are shown in Fig. 1. It can be seen that CuFe_2_O_4_ had a relatively smooth nonporous surface and reduced CuFe_2_O_4_ had fluffy appearance. The specific surface area (SSA) of CuFe_2_O_4_ and reduced CuFe_2_O_4_ was obtained from the N_2_ adsorption–desorption isotherms (Fig. S1a†). It can be seen that both the isotherms could be classified as type IV based on the IUPAC classification scheme. The SSA was measured to be 15.6 m^2^ g^−1^ for CuFe_2_O_4_ and 51.8 m^2^ g^−1^ for reduced CuFe_2_O_4_, respectively. The pore size distribution showed that there was a significant increase in volume of pores ranged from 2 to 8 nm after H_2_ reduction (Fig. S1b†).

**Fig. 2:**
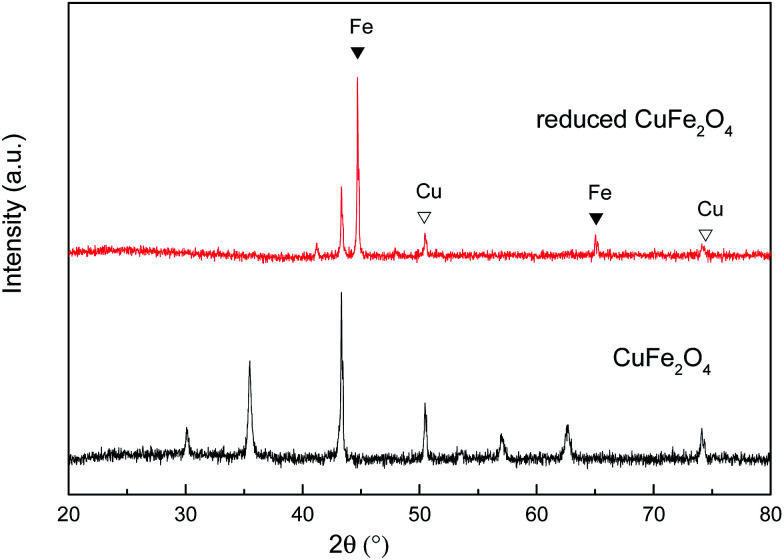
XRD patterns of CuFe_2_O_4_ and reduced CuFe_2_O_4_.

The X-ray diffraction patterns of CuFe_2_O_4_ and reduced CuFe_2_O_4_ are presented in Fig. 2. In the pattern of the CuFe_2_O_4_, all of the diffraction peaks matched well with the standard XRD pattern (PDF #77-0010), which indicated that the prepared CuFe_2_O_4_ had great purity. In the pattern of the reduced CuFe_2_O_4_, the (110) and (200) diffractions of Fe (PDF #99-0064) and (200) and (220) diffractions of Cu (PDF #70-3039) can be observed simultaneously, indicating the successful loading of Fe^0^ and Cu^0^ bimetallic particles in the reduced CuFe_2_O_4_.

**Fig. 3:**
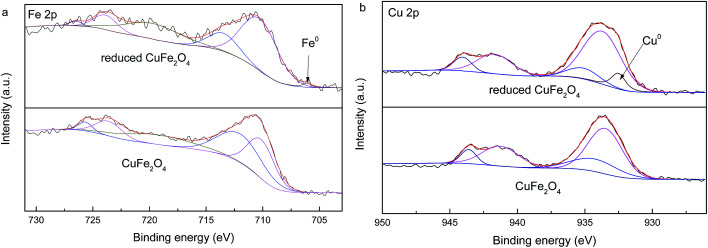
XPS spectra for Fe 2p (a) and Cu 2p (b) of CuFe_2_O_4_ and reduced CuFe_2_O_4_.

Surface elemental composition of CuFe_2_O_4_ and reduced CuFe_2_O_4_ is analyzed by the use of XPS (Fig. 3). In Fig. 3a, the binding energy at 711 eV and 725 eV can be ascribed to Fe 2p_3/2_ and Fe 2p_1/2_ according to previous study.^20^ The presence of the peak around 710.1 and 712.0 eV (for CuFe_2_O_4_) suggested that Fe^3+^ existed in two coordination environments where A-sites at higher binding energy and B-sites at lower binding energy, respectively.^21^ After redox reaction, the presence of a Fe^0^ peak with weak intensity at 706.1 eV was further evidence for the loading of Fe^0^ in the reduced CuFe_2_O_4_.^22^ For the XPS of Cu 2p regions (Fig. 3b), the peak at binding energy of 932.5 eV for the reduced CuFe_2_O_4_ was assigned to Cu^0^, which further confirmed the formation of Cu^0^ phase in the reduced CuFe_2_O_4_.^23^ The surface of the reduced CuFe_2_O_4_ samples contained 2.2% Fe^0^ and 10.6% Cu^0^ based on XPS analysis.

### 3.2. Catalytic activity of reduced CuFe_2_O_4_

**Fig. 4:**
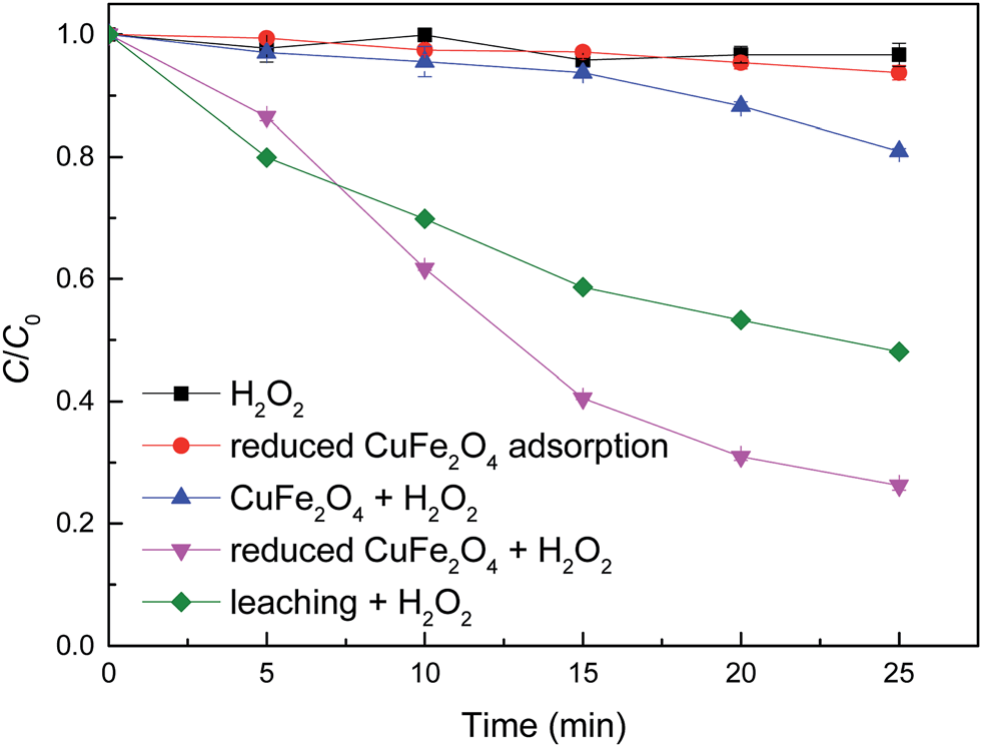
Comparison of the removal efficiency of MB with different catalytic systems. Conditions: [H_2_O_2_] = 0.5 mM, catalyst dosage = 0.1 g L^−1^, [MB] = 50 mg L^−1^, initial pH = 3.2 ± 0.1.

Batch experiments were conducted to compare the removal efficiencies of MB by various processes. As shown in Fig. 4, CuFe_2_O_4_ exhibited relatively low catalytic activity and only 20% of MB was decoloured after 25 min. By contrast, MB was rapidly degraded by reduced CuFe_2_O_4_ and greater than 74% of MB was destructed within 25 min at 0.5 mM H_2_O_2_ and 0.1 g L^−1^ catalyst dosage. Meanwhile, H_2_O_2_ alone showed no remarkable degradation of MB and less than 7% of MB was adsorbed onto reduced CuFe_2_O_4_. The kinetic data were then fitted to a pseudo-first-order kinetic model (*C* = *C*_0_e^−*kt*^). The Fenton-like reaction rate *k* (min^−1^) was calculated to be 0.007 (*R*^2^ = 0.87) and 0.055 (*R*^2^ = 0.98) min^−1^ for CuFe_2_O_4_ and reduced CuFe_2_O_4_, respectively. These results clearly indicate that the catalytic activity of CuFe_2_O_4_ is significantly enhanced after H_2_ reduction. One possible reason was the increased SSA, which provided more active sites for H_2_O_2_ decomposition and produced more reactive oxidants such as ˙OH. The other possible reason was the introduction of Fe^0^ and Cu^0^, which could facilitate the decomposition of H_2_O_2_ into ˙OH and accelerate electron transfer from Fe^0^ and/or Cu^0^ to CuFe_2_O_4_.^16,24^

To better understand the contribution of homogeneous Fenton reaction catalyzed by the leaching Fe and Cu ions on the MB degradation, experiment in homogeneous systems was carried out by removing reduced CuFe_2_O_4_ catalyst after vigorous agitation for 25 min. As shown in Fig. 4, the removal of MB after a reaction period of 25 min in the homogeneous Fenton-like reaction system was 52%. By contrast, the removal of MB catalyzed by reduced CuFe_2_O_4_ at 25 min was 74%. These results suggest that the removal of MB was attributed by both the homogeneous and heterogeneous Fenton-like reactions. Similar results were also obtained by Fenton-like degradation of 2,4-dichlorophenol using Fe_3_O_4_ magnetic nanoparticles, which assumed that the removal of 2,4-dichlorophenol was partially attributed to the bulk homogeneous Fenton reaction due to the dissolved Fe ions.^6^ The leached ions were determined in our study and the concentrations of total dissolved Fe and Cu were 0.49 and 1.09 mg L^−1^ after a reaction period of 25 min.

A comparison was carried out between the reaction rate constant of reduced CuFe_2_O_4_ with those reported in previous studies.^25–31^ Based on the obtained results (Table S1†), it seems that the proposed heterogeneous Fenton-like system leads to a high efficiency for MB degradation.

### 3.3. Effects of parameters on MB degradation

The degradation of organic pollutants was usually influenced by pH, catalyst dosage and H_2_O_2_ concentration. The degradation of MB over time under different experimental conditions was evaluated.

**Fig. 5:**
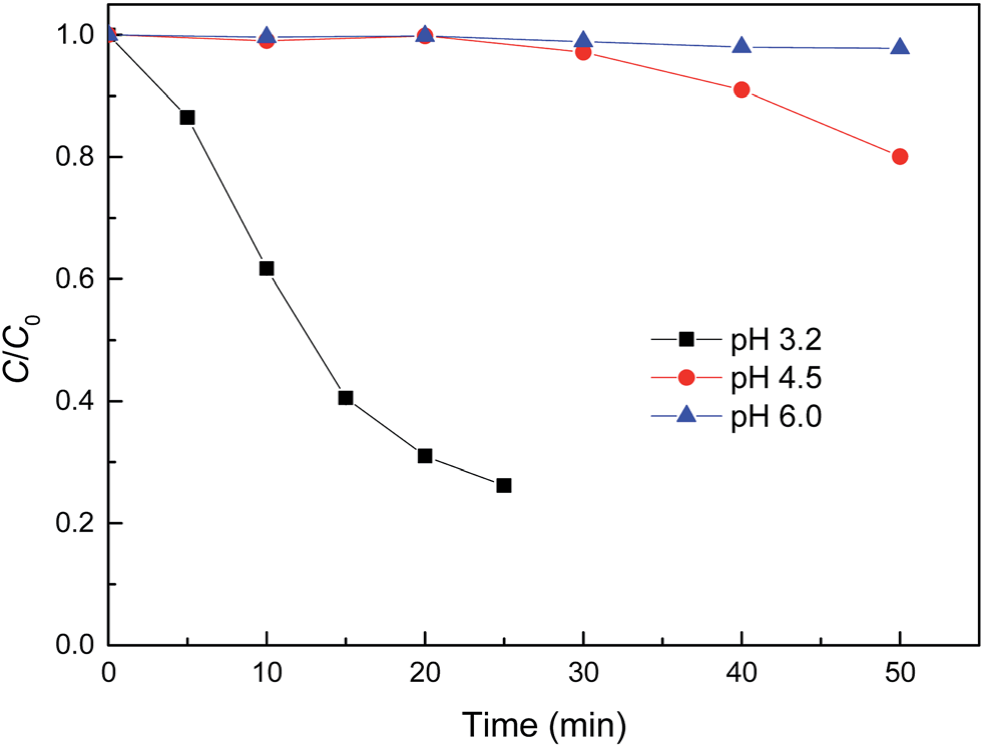
Effect of initial pH on MB degradation by reduced CuFe_2_O_4_. Conditions: [H_2_O_2_] = 0.5 mM, [reduced CuFe_2_O_4_] = 0.1 g L^−1^, [MB] = 50 mg L^−1^.

Fig. 5 shows the effect of pH on the removal of MB by reduced CuFe_2_O_4_. It can be seen that pH had a distinct influence on the removal of MB by Fenton-like reaction. The relatively slow degradation of MB was observed at pH values of 4.5 and 6.0, while a lower pH induced a higher kinetic rate. The increased oxidation efficiency at lower pH values can be attributed to the higher oxidation potential of ˙OH and the more dissolved fraction of iron species.^12^ It was also proposed that acidic conditions were favourable for the stability of H_2_O_2_ and were beneficial for the generation of ˙OH and the formation of metal oxide-pollutant inner–sphere complexes that will promote reaction.^6^ On the other hand, as Fe^0^ and Cu^0^ were loaded on the surface of reduced CuFe_2_O_4_, the lower pH will favor the generation of Fe^2+^ and Cu^+^ (eqn (1)–(3)), which could promote the decomposition of H_2_O_2_ into ˙OH (eqn (4) and (6)).^32^

**Fig. 6:**
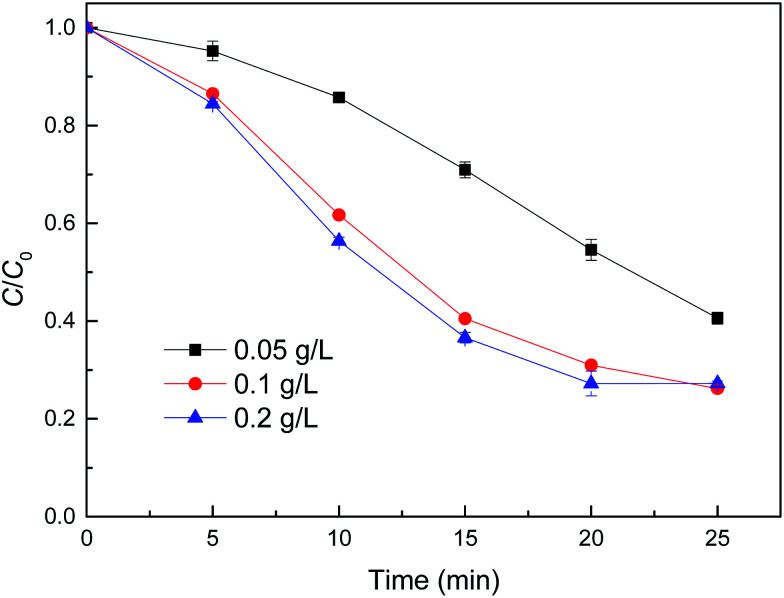
Effect of catalyst dose on MB degradation by reduced CuFe_2_O_4_. Conditions: [H_2_O_2_] = 0.5 mM, [MB] = 50 mg L^−1^, initial pH = 3.2 ± 0.1.

Fig. 6 shows the degradation of MB by reduced CuFe_2_O_4_ at different catalyst dosages. It can be observed that the removal of MB by degradation increased along with increasing catalyst dosages. The degradation of MB achieved 59.4% with 0.05 g L^−1^ catalyst dosage, and up to 73.8% when the catalyst dosage increased to 0.1 g L^−1^. This finding was likely attributed to the increased amount of active sites on the solid catalyst surface, which was expected to accelerate the reactions of H_2_O_2_ decomposition. Moreover, increasing catalyst dosage could result in a higher iron dissolution, and ultimately producing more radicals. However, when reduced CuFe_2_O_4_ addition increased to 0.2 g L^−1^, the degradation of MB was not further enhanced, probably due to the agglomeration of particles and the scavenging of ˙OH or other radicals by present iron species through undesirable reactions.^12^

**Fig. 7:**
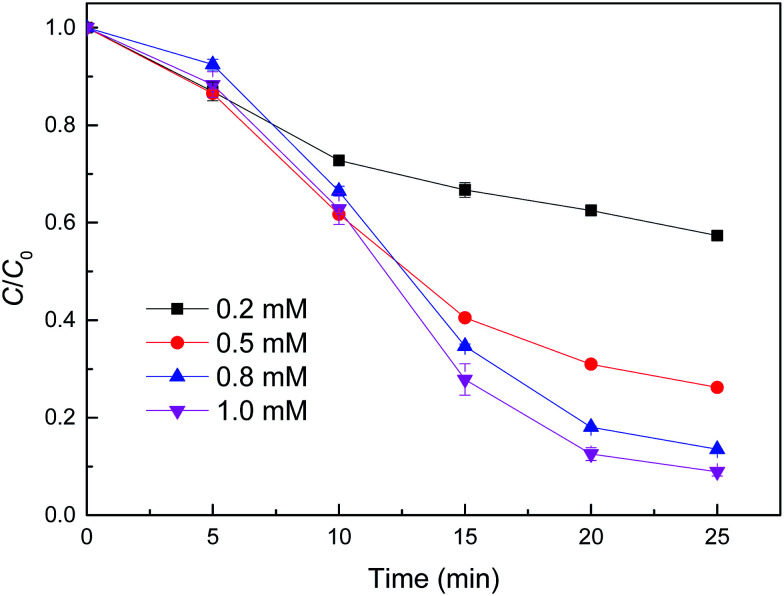
Effect of H_2_O_2_ concentration on MB degradation by reduced CuFe_2_O_4_. Conditions: [reduced CuFe_2_O_4_] = 0.1 g L^−1^, [MB] = 50 mg L^−1^, initial pH = 3.2 ± 0.1.

The effect of H_2_O_2_ concentration on the removal of MB using reduced CuFe_2_O_4_ was also investigated (Fig. 7). It was observed clearly that the degradation of MB was gradually accelerated with H_2_O_2_ concentration increasing from 0.2 to 1.0 mM. This positive correlation could be related to the accelerated generation of oxidizing intermediates that were responsible for MB oxidation, when considering that H_2_O_2_ alone had a negligible degradation effect on MB. It was proposed that H_2_O_2_ is the precursor in the reaction with ≡Fe^2+^ generating ˙OH.^33^ With sufficient H_2_O_2_, the amount of ˙OH generated will be enhanced, leading to a high removal efficiency of MB.

### 3.4. Stability and reusability of reduced CuFe_2_O_4_

**Fig. 8:**
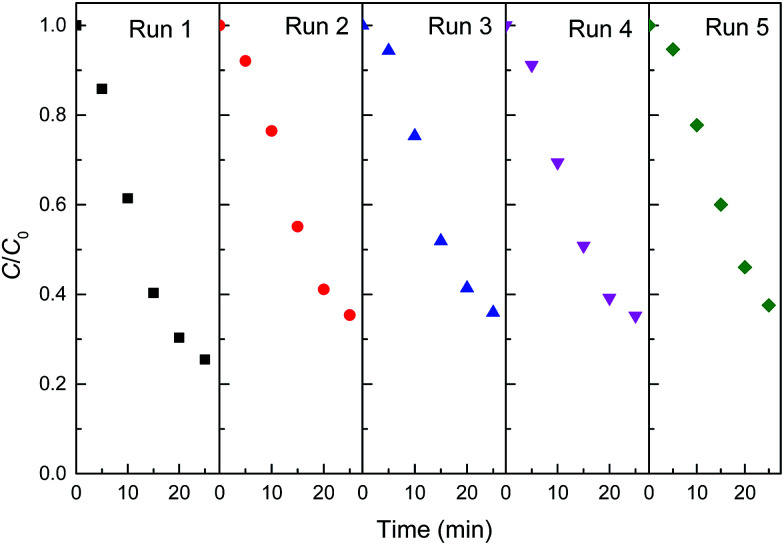
Degradation of MB in different batch runs in the reduced CuFe_2_O_4_/H_2_O_2_ system.

Stability is an important factor for the application of catalyst in heterogeneous Fenton-like reaction. From the view of practical application, the long term stability of reduced CuFe_2_O_4_ is crucial. Therefore, the leaching characteristic and the activity variation of reduced CuFe_2_O_4_ in cycles are especially concerned. The leaching ions were determined and the concentration of total dissolved iron and copper were 0.49 and 1.09 mg L^−1^ after 25 min reaction. This phenomenon partially confirmed the stability of catalyst. Successive experiments were performed to evaluate the possibility of reduced CuFe_2_O_4_ reuse. As shown in Fig. 8, it was observed that reduced CuFe_2_O_4_ remained a high catalytic activity after five consecutive runs. The XRD pattern of the catalyst after five cycles showed that crystal phase of the used catalyst was almost the same as that of the fresh catalyst (Fig. S2†). These results demonstrate that reduced CuFe_2_O_4_ has a high stability and a good reusability after the recycling tests, suggesting the feasibility of the reduced CuFe_2_O_4_/H_2_O_2_ system for a longer reaction time.

**Fig. 9:**
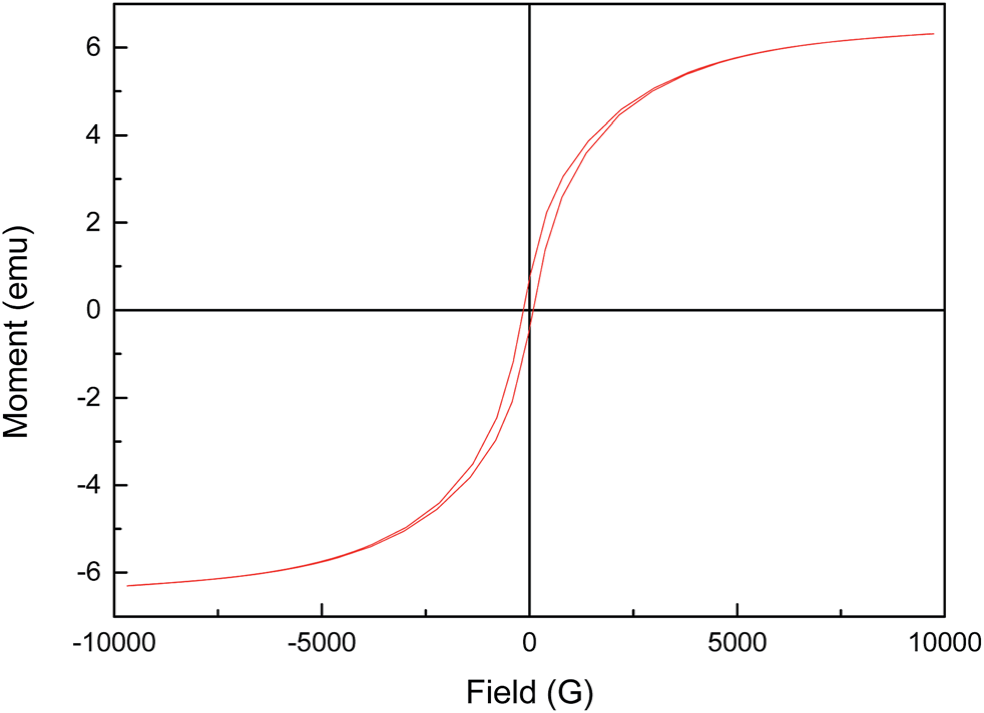
Magnetization curve of reduced CuFe_2_O_4_.

The magnetization curves of reduced CuFe_2_O_4_ were investigated by vibrating sample magnetometer (VSM) at 25 °C and the results were illustrated in Fig. 9. The magnetic hysteresis curve revealed that reduced CuFe_2_O_4_ was ferromagnetic and had a magnetic saturation of about 6.3 emu g^−1^, which ensured that the catalyst could be easily separated by a magnet and reused from aqueous solution.

### 3.5. The enhanced reaction mechanism

**Fig. 10:**
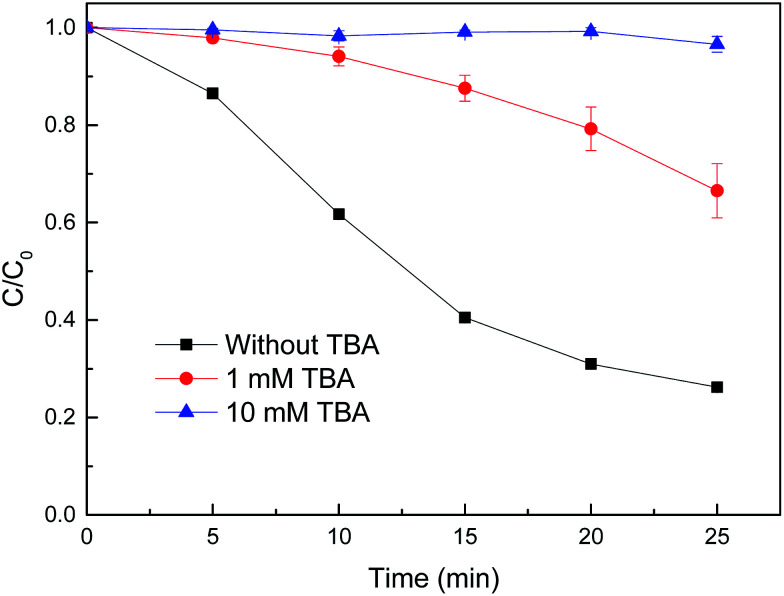
Effect of TBA on MB degradation by reduced CuFe_2_O_4_. Conditions: [H_2_O_2_] = 0.5 mM, [reduced CuFe_2_O_4_] = 0.1 g L^−1^, [MB] = 50 mg L^−1^, initial pH = 3.2 ± 0.1.

In order to discriminate the active species in the reduced CuFe_2_O_4_/H_2_O_2_ system for MB degradation, *tert*-butyl alcohol (TBA) was used as the scavenger of ˙OH in this study. As shown in Fig. 10, the degradation efficiency of MB decreased from 75% without inhibitor to 34 and 3% with the addition of 1 and 10 mM TBA, respectively. There was almost no MB degradation with the addition of 10 mM TBA during the reaction, indicating that the ˙OH produced by H_2_O_2_ in the Fenton-like reaction was scavenged. These results indicate that MB is mainly decomposed by the attack of ˙OH.

**Fig. 11:**
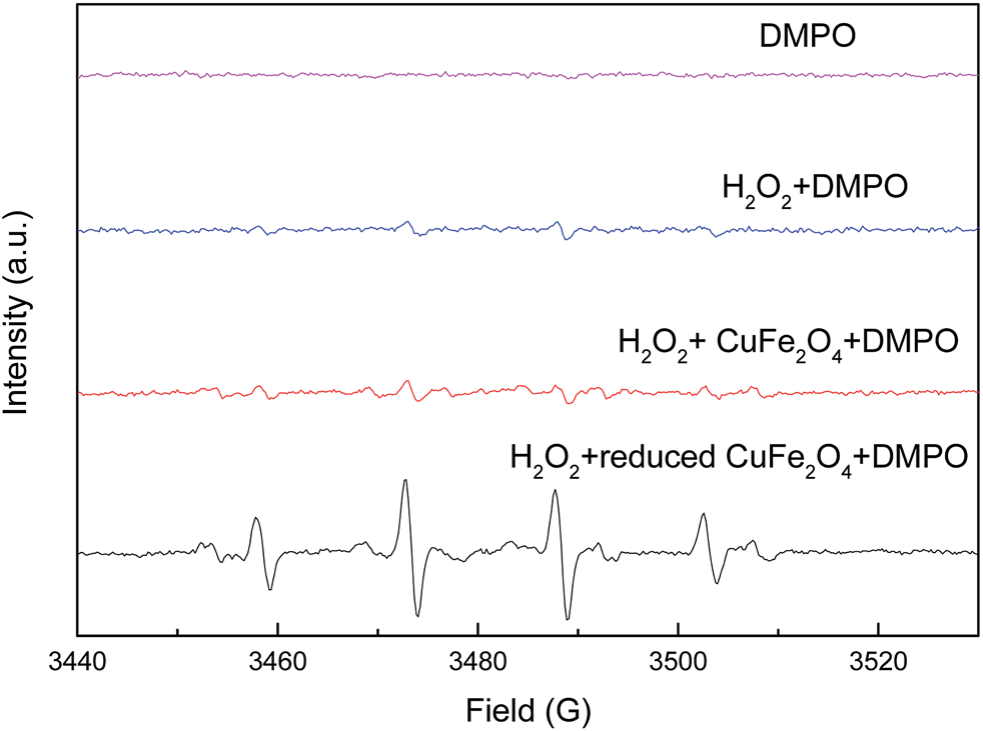
DMPO spin trapping ESR spectra of ˙OH in the systems.

To ascertain the reaction mechanism, electron spin resonance (ESR) spectroscopy was performed by using 5,5-dimethyl-1-pyrroline-*N*-oxide (DMPO) as trapping agent to examine ˙OH produced in the heterogeneous Fenton-like reaction. As shown in Fig. 11, the ESR spectrum in the presence of catalysts displayed a 4-fold characteristic peak of DMPO–˙OH adduct with an intensity ratio of 1 : 2 : 2 : 1. The intensity of DMPO–˙OH peaks by using reduced CuFe_2_O_4_ as catalyst was much stronger than CuFe_2_O_4_, demonstrating a high catalytic activity in the reduced CuFe_2_O_4_/H_2_O_2_ system. Therefore, it can be concluded that reduced CuFe_2_O_4_ could effectively activate H_2_O_2_ to generate ˙OH and the ˙OH was the predominant active radical in the heterogeneous Fenton-like reaction.

The above results have shown that the ˙OH is the major active radical in the heterogeneous Fenton-like processes. Moreover, the enhanced catalytic activity is displayed by catalyst of reduced CuFe_2_O_4_, which shows high MB removal efficiency. The Fenton-like reaction rate *k* (min^−1^) was calculated to be 0.007 and 0.055 min^−1^ for CuFe_2_O_4_ and reduced CuFe_2_O_4_, respectively. When normalized by SSA, the *k*/SSA values indicate a higher MB removal efficiency for the reduced CuFe_2_O_4_ than for the CuFe_2_O_4_. This suggests that Fe^0^ and Cu^0^ bimetallic particles loaded in the reduced CuFe_2_O_4_ play important role in the heterogeneous Fenton reaction. According to all above experimental results, a possible mechanism for reduced CuFe_2_O_4_ degradation of MB has been proposed. In the first step, the H_2_O_2_ molecules can be adsorbed on reduced CuFe_2_O_4_ and react with the metallic particles. Specifically, the Fe^0^ can be oxidized to Fe^2+^*via* a two electron transfer (eqn (2)) and Cu^0^ can be oxidized to Cu^+^*via* a one electron transfer from the particles surface to H_2_O_2_ (eqn (3)).^18,34^ These oxidation reactions were further confirmed by the results of XPS (Fig. S3†). The peak of Fe^0^ disappeared after reaction. The atomic ratio of Cu^0^ decreased from 10.6% in the fresh catalyst to 4.9% in the used catalyst.

**Fig. 12:**
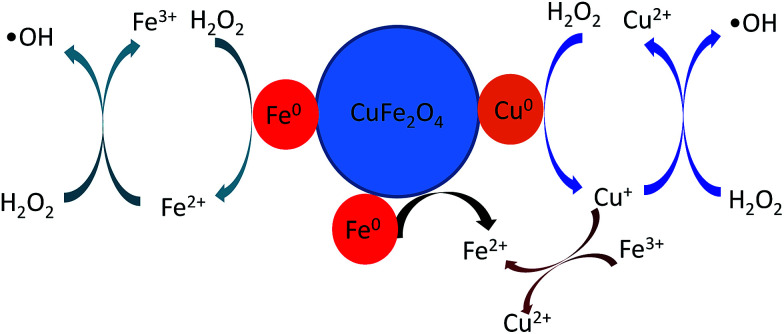
Schematic diagram of MB degradation mechanism by reduced CuFe_2_O_4_.

Then, Fe^2+^ on the surface participate in the reaction by activating H_2_O_2_ molecules to produce ˙OH according to the Haber–Weiss mechanism (eqn (4) and (5)).^33,35^ Similarly, Cu^+^ on the surface can also participate in the reaction by activating H_2_O_2_ molecules to generate ˙OH (eqn (6)).^17,18^ On the other hand, the loading of Fe^0^ could act as an electron transfer agent during reaction, which could easily reduce Fe^3+^ species in the CuFe_2_O_4_ phase to regenerate the active Fe^2+^ species (eqn (9)).^13,15^ Since the standard reduction potential of Fe^3+^/Fe^2+^ is 0.77 V and Cu^2+^/Cu^+^ is 0.17 V, the redox cycles of Fe^3+^/Fe^2+^ and Cu^2+^/Cu^+^ are also proposed (eqn (8)).^17,18^ Therefore, based on above analysis, a possible enhanced reaction mechanism of MB degradation by reduced CuFe_2_O_4_ is proposed as illustrated in Fig. 12.

## 4. Conclusions

Reduced CuFe_2_O_4_ was synthesized and used as a heterogeneous Fenton-like catalyst, which exhibited much higher catalytic activity towards the degradation of MB in the presence of H_2_O_2_ compared with raw CuFe_2_O_4_. The use of 0.1 g L^−1^ reduced CuFe_2_O_4_ induced nearly 74% of MB degradation within 25 min in the presence of 0.5 mM H_2_O_2_ at initial pH 3.2. The characterization and experimental results suggested that the high catalytic activity was attributed to the high surface area and the presence of Fe^0^/Cu^0^ bimetallic particles on the surface of reduced CuFe_2_O_4_. The reusability tests indicated that reduced CuFe_2_O_4_ was relatively stable and could be reused several times as a Fenton-like catalyst. Moreover, reduced CuFe_2_O_4_ displayed a superparamagnetic property, which allowed them to be easily separated and collected in practical applications.

## Conflicts of interest

There are no conflicts to declare.

## Supplementary Material

RA-008-C7RA12488K-s001
