# Supplementary material for: Enhanced heterogeneous Fenton-like degradation of methylene blue by reduced CuFe2O4
Source: RSC Adv. 2018 Jan 3;8(2):1071–7. doi: 10.1039/c7ra12488k (PMC9076977; doi:10.1039/c7ra12488k)
Supplement: RA-008-C7RA12488K-s001 [file RA-008-C7RA12488K-s001.pdf]

Electronic Supplementary Material (ESI) for RSC Advances.
This journal is © The Royal Society of Chemistry 2017

Enhanced heterogeneous Fenton-like degradation of methylene blue by reduced $CuFe_2O_4$

Qingdong Qin[a], Yahong Liu[a], Xuchun Li[b], Tian Sun[a], Yan Xu[a, *]

[a]School of Civil Engineering, Southeast University, Nanjing 210096, China

[b]School of Environmental Science and Engineering, Zhejiang Gongshang University, Hangzhou 310018, China

∗ Corresponding author: xuxucalmm@seu.edu.cn, Tel.: +86 25 83790757, Fax: +86 25 83790757

Table S1 Comparison of MB removal using different Fenton-like catalysts

Catalysts	Catalyst dose (g/L)	$[MB]_0$ (mg/L)	$[H_2O_2]_0$ (mM)	pH	k (min^{-1}) [a]	References
Fe_3O_4/CeO_2	1	100	163.7	6.0	0.034	[1]
rGSs/Fe_2O_3/PPy	0.5	80	~2450	6.5	0.031	[2]
Fe_3O_4@SiO_2	1.0	50	~680	6.5	0.020	[3]
FePt	0.005	5	~1140	5.5	0.023	[4]
MnO_2-coated Fe-pillared bentonite	0.1	50	176.4	2.4	0.013	[5]
Ferrocene	0.186	10	23.58	4.0	0.026	[6]
GT-Fe	1.0	50	~330	3.1	0.021	[7]
Reduced $CuFe_2O_4$	0.1	50	0.5	3.2	0.055	This work

[a] k caculated from the pseudo-first-order kinetic model ($C=C_0e^{-kt}$)

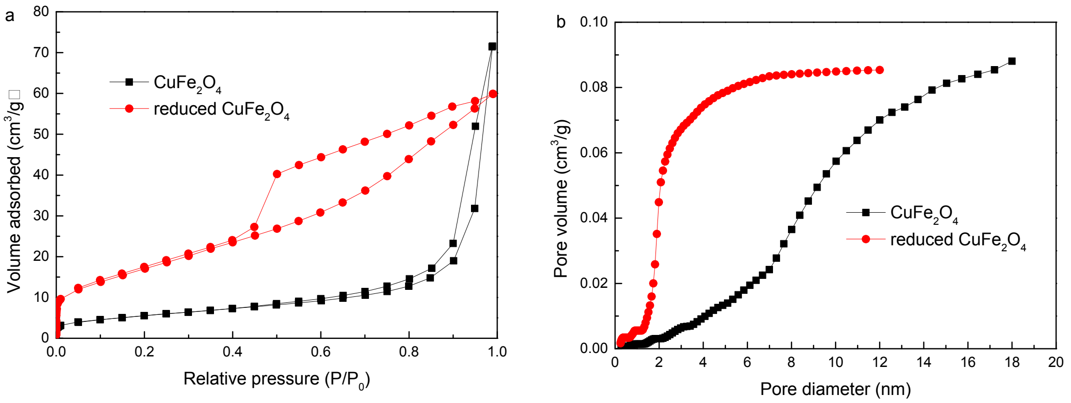

Fig.S1 N_2 adsorption isotherms (a) and pore size distribution curves (b) of $CuFe_2O_4$ and reduced $CuFe_2O_4$.

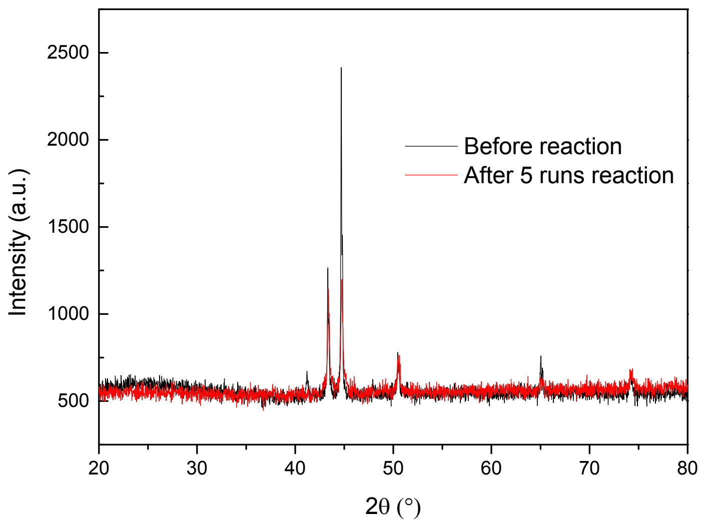

Fig.S2 XRD patterns of reduced $CuFe_2O_4$ before and after reaction.

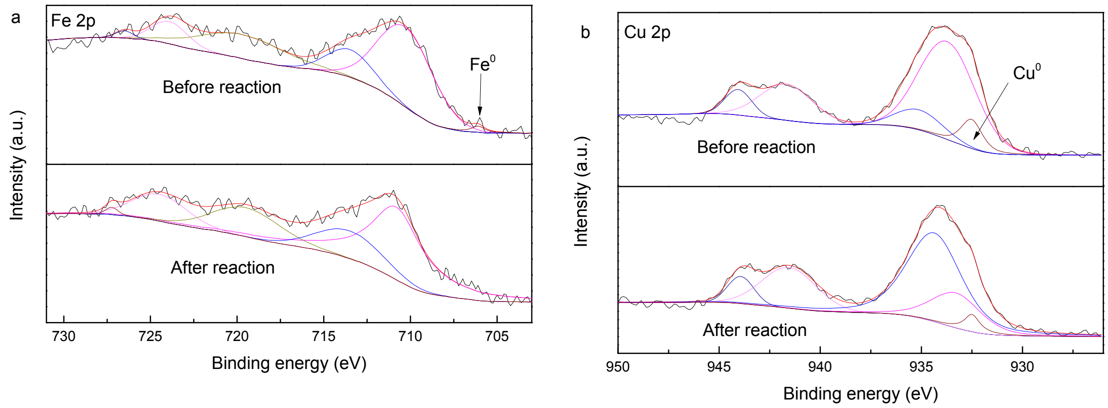

Fig.S3 XPS spectra for Fe 2p (a) and Cu 2p (b) of reduced $CuFe_2O_4$ before and after reaction.

References

1. K. Y. Li, Y. Q. Zhao, C. S. Song and X. W. Guo, *Appl. Surf. Sci.*, 2017, **425**, 526-534.
2. J. S. Zhang, T. J. Yao, C. C. Guan, N. X. Zhang, H. Zhang, X. Zhang and J. Wu, *J. Colloid Interface Sci.*, 2017, **505**, 130-138.
3. S. T. Yang, W. Zhang, J. R. Xie, R. Liao, X. L. Zhang, B. W. Yu, R. H. Wu, X. Y. Liu, H. L. Li and Z. Guo, *RSC Adv.*, 2015, **5**, 5458-5463.
4. S. C. Hsieh and P. Y. Lin, *J. Nanopart. Res.*, 2012, **14**, 10.
5. Z. L. Shi, X. X. Wang and S. H. Yao, *Chin. J. Inorg. Chem.*, 2015, **31**, 696-702.
6. Q. Wang, S. L. Tian and P. Ning, *Ind. Eng. Chem. Res.*, 2014, **53**, 643-649.
7. T. Shahwan, S. Abu Sirriah, M. Nairat, E. Boyaci, A. E. Eroglu, T. B. Scott and K. R. Hallam, *Chem. Eng. J.*, 2011, **172**, 258-266.
